# Anatomic measurements of cerebral venous sinuses in idiopathic intracranial hypertension patients

**DOI:** 10.1371/journal.pone.0196275

**Published:** 2018-06-01

**Authors:** Srikanth R. Boddu, Pierre Gobin, Cristiano Oliveira, Marc Dinkin, Athos Patsalides

**Affiliations:** 1 Division of Interventional Neuroradiology, Department of Neurological surgery, New York Presbyterian Hospital / Weill Cornell Medical Center, New York, NY, United States of America; 2 Interventional Neuroradiology, New York Presbyterian Queens Hospital, Flushing, NY, United States of America; 3 Department of Ophthalmology, New York Presbyterian Hospital, Weill Cornell Medical College, New York, NY, United States of America; Universitatsklinikum Freiburg, GERMANY

## Abstract

**Purpose:**

Magnetic resonance venography (MRV) has not been validated in pre-operative planning of the dural venous sinus stenting (VSS) among idiopathic intracranial hypertension (IIH) patients. We aim to prospectively evaluate dural venous sinus measurement in IIH patient population on two-dimensional time-of-flight (2D-TOF) MRV and Three-dimensional contrast-enhanced (3D-CE) MRV acquisitions and compare them against real-time endoluminal measurements with intravascular ultrasound (IVUS), served as the reference.

**Materials and methods:**

The study has been approved by the Weill Cornell Medicine institutional review board. All patients signed written informed consent approved by IRB. Prospective evaluation of forty-five consecutive IIH patients treated with VSS at our institution were evaluated. Patients with pre-stent magnetic resonance venography (MRV) ≤ 6-months of VSS and intravascular ultrasound (IVUS) during VSS constituted the study population. Maximum diameter (in mm), Area (in cm2) and Perimeter (in cm) were measured at posterior 1/3rd of superior sagittal sinus (SSS), proximal transverse sinus (PTS), proximal sigmoid sinus (PSS) and mid sigmoid sinus (MSS) on 2D-TOF-MRV, 3D-CE-MRV and IVUS. Statistical analysis performed using box and whisker plots, Bland-Altman analysis and paired sample t-test.

**Results:**

Twenty (n = 20) patients constituted our study population. The mean age was 30±11 years (7–59 years) and 18 out of 20 were female patients. Mean weight and BMI (range) were 86.3±18.3 kilograms (30.8–107.5 kgs) and 32.9±6.8 kg/M2 (16.4–48.3kg/M2) respectively. The CE-MRV significantly oversized the cerebral venous sinuses compared to TOF-MRV (Dmax: +2.0±1.35 mm, p<0.001; Area: +13.31±10.92 mm2, p<0.001 and Perimeter: +4.79±3.4 mm, p<0.001) and IVUS (Dmax: +1.52±2.16 mm, p<0.001; Area: +10.03±21.5 mm2, p<0.001 and Perimeter: +4.15±3.27 mm, p<0.001). The TOF-MRV sinus measurements were in good agreement with the IVUS measurements with no significant variation (Dmax: +.21±2.23 mm, p = 0.49; Area: +2.51±20.41mm2, p = 0.347 and Perimeter: +.001±1.11 mm, p = 0.991).

**Conclusion:**

We report baseline cerebral venous sinus measurements (maximum diameter, area and perimeter) in patients with idiopathic intracranial hypertension. In our experience, TOF-MRV is a reliable representation of endoluminal cerebral venous sinus dimensions, and CE-MRV measurements reflected an overestimation of the endoluminal sinus dimensions when compared against the real time IVUS measurements.

## Introduction

Venous sinus stenting (VSS) has become an effective treatment choice for refractory idiopathic intracranial hypertension (IIH).[1] Accurate measurement of the venous sinuses is crucial for choosing the appropriate stent size. Oversize stents may result in focal ipsilateral headaches due to superimposed dural stretching/ irritation and a spurious proximal narrowing of the sinus adjacent to the stent due to inherent collapsible nature of cerebral venous sinuses.[2] In contrary, undersized stents may result in incomplete recanalization of stenosis and stent migration.

Magnetic resonance venography (MRV) is a widely accepted modality for the diagnosis of venous sinus stenosis in patients with idiopathic intracranial hypertension (IIH).[3–5] MRV is non-invasive, offers three-dimensional reconstructions, and does not require exposure to ionizing radiation or iodinated contrast media. However, use of MRV for sinus measurements has not been validated for the measurement of venous sinus dimensions for pre-operative planning of the dural venous sinus stent placement. The evaluation of dural venous sinuses on MRV is predominantly subjective based on the reader’s impression in a descriptive method with no established quantitative method.[6] The variation of sinus measurements on different techniques of magnetic resonance venography (MRV) is unknown leaving an uncertainty of which MRV acquisition should be used for stent size selection.

Catheter venography (CV), the presumed gold standard for determining vascular anatomy, is primarily dependent on luminal opacification with limited potential to detect subtle intraluminal structures or wall lesions, including thrombus, intraluminal valves, septa, and flaps.[7] CV not only limits an overall evaluation of endoluminal structures due to its uni/bi-planar visualization[8], also found to be less sensitive in revealing the exact nature of narrowed vein segments.[7] Luminal measurements on the catheter venography are vulnerable to measurement errors due to the uni/biplanar evaluation of the triangular dural venous sinuses.

The purpose of this study was to prospectively evaluate dural venous sinus measurement in an IIH patient population on 2D-TOF MRV and 3D CE-MRV acquisitions and compare them against real-time endoluminal measurements with duplex intravascular ultrasound (IVUS). IVUS with real-time 360^0^ panoramic endoluminal visualization capability of the venous sinuses and simultaneous location confirmation on fluoroscopy served as the reference.

## Materials and methods

### Patient selection

The study has been approved by the Weill Cornell Medicine institutional review board. All patients signed written informed consent approved by IRB. This is a prospectively collected data of the patients who underwent venous sinus stenting (VSS) at our institution. The patients are enrolled either as a part of ongoing FDA approved clinical trial “Venous Sinus Stenting for Idiopathic Intracranial Hypertension Refractory to Medical Therapy (ClinicalTrials.gov Identifier: NCT01407809) or in a prospective patient registry, both approved by our Institutional Review Board. Forty-five consecutive idiopathic intracranial hypertension (IIH) patients treated with venous sinus stenting (VSS) were prospectively evaluated. Patients with pre-stent magnetic resonance venography (MRV) ≤ 6-months of VSS and intravascular ultrasound (IVUS) during VSS constituted the study population.

### Study parameters

The anatomic measurements of the cerebral venous sinuses were measured using Magnetic resonance venography (MRV) and IntraVascular UltraSound (IVUS).

Four locations of cerebral venous sinuses (Posterior 1/3^rd^ of superior sagittal sinus [SSS], Proximal transverse sinus [PTS], Proximal sigmoid sinus [PSS] and Mid sigmoid sinus [MSS)]) were evaluated on three imaging acquisition techniques (2D-TOF-MRV, 3D CE-MRV and IVUS), using three measurements at each location (Maximum diameter [D_max_], Area and Perimeter). Four sets of measurements were obtained for each image acquisition technique with each measurement set containing: Maximum diameter along axis (in mm), Area of the sinus (in cm^2^) and Perimeter of the sinus (in cm).

#### Magnetic resonance venography (MRV)

All MRVs were performed on 1.5-T scanners using a contiguous 2D time-of-flight (TOF) MR angiographic technique and an inferior saturation band to eliminate signal from arterial structures. Sections with thickness: 1.8 mm and space: 1.8mm were acquired in the coronal plane using the following parameters: 20/4.1 (TR/TE), 70° flip angle, Nex:1, 210-mm field of view, and 320 × 192 matrix. Post processing of the source images was performed using the maximum (pixel) intensity projection (MIP) method, generating 12 MIP projections at 15° increments.

Contrast enhanced (CE) MRV was performed using 3D T1-fast spoiled gradient-echo pulse sequence with TR/TE = 11/2.3 ms, flip angle = 25°, FOV = 25 cm, 256 × 256 sampling matrix, 120-axial acquisitions with slice thickness: 1.5 mm and space: 1.5mm following 7–10 ml of intravenous gadolinium contrast dose. Post processing of the source images in coronal and sagittal reformats was performed using retrograde 50% overlap resulting in a 0.8 mm slice thickness.

**Fig 1:**
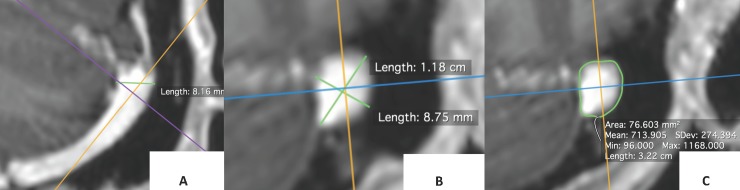
Multiplanar curved reformats of the proximal sigmoid sinus performed on ADW workstation to produce optimal venous sinus visualization in axial and coronal planes or measurement of maximum diameter (A & B) and area (C).

**Fig 2:**
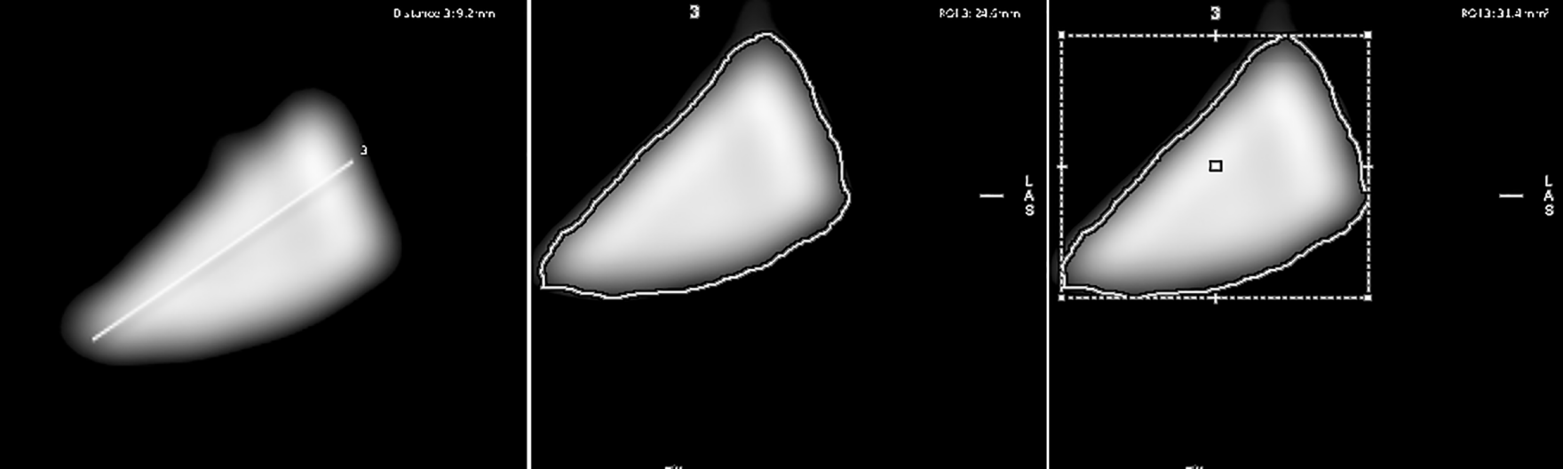
End-on luminal view of superior sagittal sinus (SSS) generated by the ADW software for measurement of the maximum diameter (A), perimeter (B) and area (C).

Post processing of the DICOM source images was performed using three-dimensional multi-planar curved reformats on advanced workstation (ADW: 4.7) for optimal visualization of maximum luminal diameter [Fig 1]. Individual anatomic measurements were recorded on the automated end-on luminal view generated by the ADW software [Fig 2].

#### Intravascular ultrasound (IVUS)

**Fig 3:**
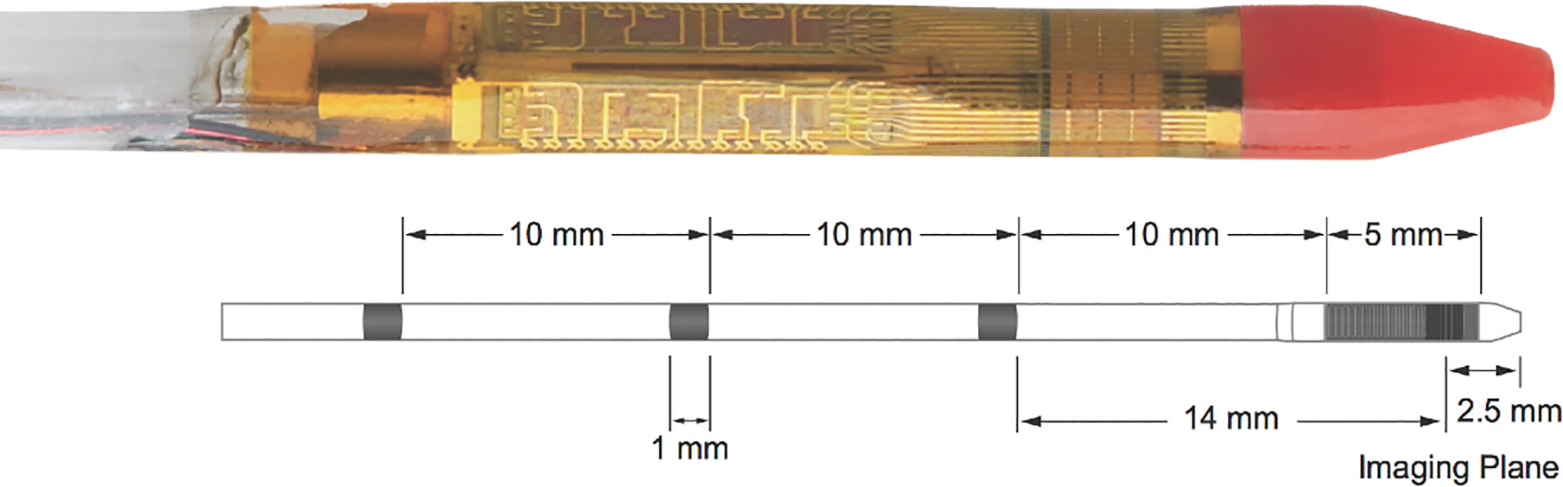
Intravascular ultrasound (IVUS) system demonstration. The system contains radiopaque markers at 10mm proximally and one 14 mm marker distally which helps to assess the length of stenosis segment. Distal 5mm ultrasound probe, which acquires the images, is located 2.5mm behind the catheter tip.

**Fig 4:**
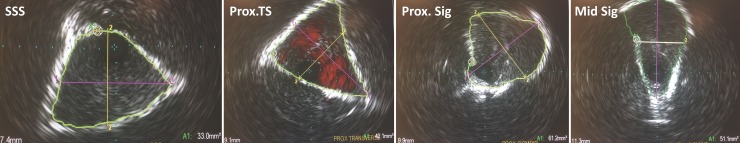
IVUS measurements at different sinus locations. The hyperechoic boundaries represent the margins of the sinuses.

IVUS was performed as a part of cerebral venography under general anesthesia using the Volcano Eagle Eye gold catheter (Volcano Corporation, Rancho Cordova, CA, USA). The 3.4 Fr IVUS catheter [Fig 3] was inserted from a transfemoral venous access via 6F guide catheter placed in the proximal internal jugular vein. The IVUS probe has 20 MHz frequency with a 40mm pro short 10.5mm transducer-to-tip distance with a 16mm field of view. The disposable IVUS electronic catheter was navigated beyond the stenosis in the transverse-sigmoid junction over the 0.014” heavy-duty wire in a monorail fashion and positioned in the posterior third of superior sagittal sinus. The IVUS catheter was then connected to the Sonosite (SonoSite Inc., Bothell, WA, USA) monitor for real time evaluation. The intraluminal images with and without color doppler were acquired during steady withdrawal of IVUS catheter using fluoroscopic guided spatial correlation [Fig 4]. The anatomic measurements were obtained using inbuilt calipers and ROI (Region of Interest) tools of the software.

#### Statistical analysis

Statistical analysis performed using SPSS version 24. Continuous variables of anatomic measurements (maximum diameter, area and perimeter) were described as mean and standard deviation (SD). The variation of the median and quartiles of 2D-TOF MRV and 3D-CE MRV measurements were reported with box and whisker plots. The bias (mean difference) and variability (SD of the differences) between the 2D-TOF MRV, 3D-CE MRV and IVUS was obtained with the Bland-Altman analysis.[9] Significance of variation between the measurements of imaging techniques was evaluated with paired sample t-test. Precision of the diagnostic parameters was presented using a 95% confidence interval. P values below 0.05 were considered significant.

## Results

Based on the inclusion and exclusion criteria twenty (n = 20) patients constituted our study population. The mean age was 30±11 years (7–59 years) and 18 out of 20 were female patients. Mean weight and BMI (range) were 86.3±18.3 kilograms (30.8–107.5 kgs) and 32.9±6.8 kg/M^2^ (16.4–48.3kg/M^2^) respectively.

**Fig 5:**
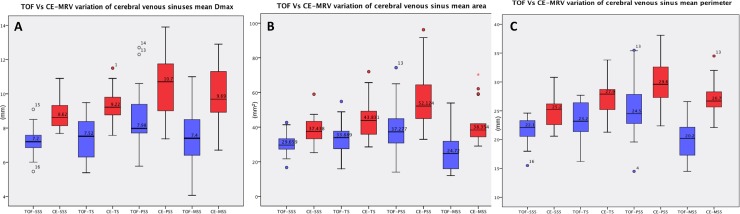
2D-TOF MRV versus 3D-CE MRV comparison of the median and interquartile ranges of dural venous sinuses. A: Mean Dmax (Maximum sinus diameter); B: Mean sinus area and C: Mean sinus perimeter.

The mean sinus measurements (D_max_, area and perimeter), 95% confidence intervals and range of measurements based on acquisition techniques are summarized in Table 1. Comparison of the median sinus measurements and interquartile ranges between 2D-TOF MRV and 3D-CE MRV were summarized in Fig 5.

**Table 1:** Summary of mean sinus measurements (D_max_, area and perimeter), 95% confidence intervals and range of measurements based on acquisition technique.

	2D-TOF-MRV			3D-CE-MRV			IVUS		
	D_max_ (mm) (95% CI) *Range*	Area (mm^2^) (95% CI) *Range*	Perimeter (mm) (95% CI) *Range*	D_max_ (mm) (95% CI) *Range*	Area (mm^2^) (95% CI) *Range*	Perimeter (mm) (95% CI) *Range*	D_max_ (mm) (95% CI) *Range*	Area (mm^2^) (95% CI) *Range*	Perimeter (mm) (95% CI) *Range*
**SSS**	7.3±0.8 (6.8–7.6) *5*.*5–9*.*0*	30.4±6.2 (27.6–33.2) *16*.*6–42*.*6*	21.5±2.3 (20.5–22.5) *15*.*5–24*.*6*	8.8±0.8 (8.4–9.2) *7*.*7–10*.*9*	38.6±8.1 (35–42.3) *25*.*3–59*	24.8±2.5 (23.7–26) *20*.*6–30*.*8*	7.6±1.3 (7.0–8.2) *5*.*8–10*	33.1±8.9 (29–37.2) *20*.*8–61*	20.3±2.5 (19.2–21.4) 14–25.3
**PTS**	7.5±1.2 (6.9–8.0) *5*.*4–9*.*5*	33.8±9.8 (29.4–38.2) *15*.*9–54*.*8*	23±3.4 (21.5–24.5) *16*.*2–27*.*7*	9.4±1.1 (8.9–9.8) *7*.*6–11*.*5*	45.1±11.5 (40–50.2) *28*.*6–72*	27.3±3.3 (25.8–28.7) *21*.*3–33*.*8*	8.1±1.4 (7.5–8.7) *6*.*1–11*.*5*	35.2±14.9 (28.7–41.7) *17*.*2–84*.*6*	21.5±3.4 (20–23) 14.7–26.8
**PSS**	8.6±1.8 (7.8–9.4) *5*.*8–12*.*7*	40±15.6 (32.9–47) *14–74*.*3*	*25*.*1*±5.1 (22.8–27.3) 1*4*.*5–35*.*4*	10.5±1.8 (9.7–11.3) *7*.*4–13*.*9*	57.7±17.8 (49.7–65.7) *32*.*9–96*.*2*	30±4.2 (28.1–31.9) *22*.*4–38*.*1*	8.3±1.8 (7.6–9.1) *5*.*8–12*.*6*	42.1±17.3 (34.5–49.6) *24*.*4–94*	23.1±4.9 (20.1–25.2) 13–34
**MSS**	7.3±1.6 (6.6–8.1) *4*.*1–11*	25.5±11.3 (20.5–30.6) *12–53*.*9*	20.4±4.2 (18.5–22.2) *14*.*5–30*.*3*	10±1.7 (9.3–10.7) *6*.*7–12*.*9*	41.5±12.1 (36.1–47) *29–70*.*3*	27.1±3.1 (25.7–28.5) *22*.*1–34*.*5*	7.6±1.4 (6.2–8.3) *4*.*3–10*.*8*	27.4±10.3 (23.5–32.4) *14*.*6–56*.*9*	19.4±4.1 (17.2–21.4) *13*.*5–29*.*8*

SSS: Superior sagittal sinus; PTS: Proximal transverse sinus; PSS: Proximal sigmoid sinus; MSS: Mid sigmoid sinus; Dmax: Maximum diameter; TOF-MRV: Time-of-flight magnetic resonance venography; CE-MRV: Contrast enhanced magnetic resonance venography; IVUS: Intravascular ultrasound.

**Fig 6:**
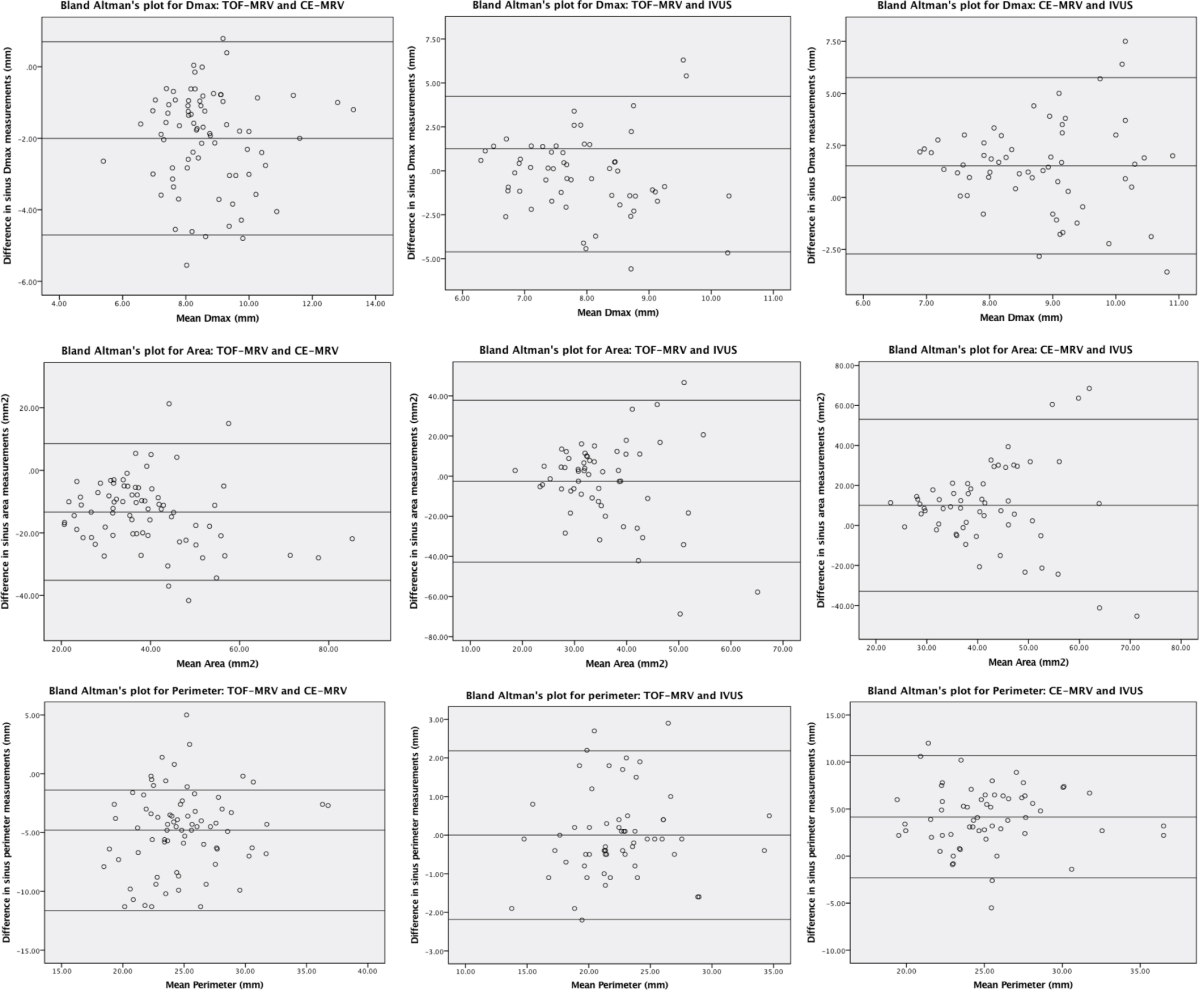
Bland—Altman’s plots evaluating bias and variability of sinus measurements (Dmax, area and perimeter) between 2D-TOF MRV, 3D-CE MRV and IVUS.

The bias (mean difference) and variability (SD of the differences) between the 2D-TOF MRV, 3D-CE MRV and IVUS measurements based on the Bland—Altman analysis and the significance of bias calculated from paired sample t-test were summarized in Table 2. The Bland—Altman’s plots evaluating the three techniques are shown in Fig 6.

**Table 2:** Summary of mean difference ± standard deviation (SD) of overall cerebral venous sinus measurements on 2D-TOF MRV, 3D-CE MRV and IVUS evaluated using Bland-Altman analysis.

	TOF-MRV Vs IVUS	CE-MRV Vs IVUS	TOF-MRV Vs CE-MRV
**D**_**max**_ (mm)	-.21±2.23 *(p = 0*.*49)*	1.52±2.16 *(p<0*.*001)*	-2.0±1.35 *(p<0*.*001)*
**Area** (mm^2^)	-2.51±20.41 *(p = 0*.*347)*	10.03±21.5 *(p = 0*.*001)*	-13.31±10.92 *(p<0*.*001)*
**Perimeter** (mm)	.001±1.11 *(p = 0*.*991)*	4.15±3.27 *(p<0*.*001)*	-4.79±3.41 *(p<0*.*001)*

Dmax: Maximum diameter; TOF-MRV: Time-of-flight magnetic resonance venography; CE-MRV: Contrast enhanced magnetic resonance venography; IVUS: Intravascular ultrasound.

## Discussion

We report the systematic anatomic measurements (maximum diameter, area and perimeter) of the cerebral venous sinuses using 2D time of flight (TOF) MRV, 3D contrast enhanced (CE) MRV and intravascular ultrasound (IVUS) techniques in patients with idiopathic intracranial hypertension (IIH). In our cohort, the CE-MRV significantly oversized the cerebral venous sinuses compared to TOF-MRV (p<0.001) and IVUS (p<0.001). The TOF-MRV sinus measurements were in good agreement with the IVUS measurements with no significant variation.

Intravenous ultrasound (IVUS) is a catheter based intravascular ultrasound technique, offers a unique three dimensional, real-time, 360^0^ endovascular visualization of the lumen as well as vessel wall irrespective of the venous sinus configuration or projection angle. Despite its conventional reputation as a gold-standard investigation, catheter venography is prone for false-positive and false-negative results in the evaluation of venous sinus stenosis. This is due to high collateral flow entering the venous sinus and reducing the degree of opacification of the sinus or from a poor position of the catheter tip where contrast material is injected preferentially into a collateral channel, resulting in suboptimal opacification of the sinus.[7,10] Sclafani and Lugli et al have reported the superiority of IVUS over catheter venography in the evaluation of patients with chronic cerebrospinal venous insufficiency.[10,11]

The 2-D TOF MRV has excellent sensitivity to slow flow and rely mainly on flow-related enhancement for producing vascular images. Flow gaps mainly related to artifacts resulting from slow intravascular blood flow, in-plane flow, and complex blood flow patterns is the main limitation of the TOF MRV.[4,5,12] With small slice thickness on the order of 1.0–1.5 mm, perpendicular plane of image acquisition to the region of interest and minimum through-plane velocity (V = d/TR, where V is the minimum velocity of flowing blood [cm/s], TR is the pulse repetition time [in ms], and d is the slice thickness [in mm]) of approximately 3 cm/s, rarely becomes an issue with in actual practice of MR venography.[13] Contrast enhanced MRV with simultaneous administration of intravenous gadolinium chelated compounds is an effective alternative to overcome this limitation. The flow signal on the TOF MRV is entirely confined to the vein lumen. In contrary, the enhancement on contrast enhanced MRV is a combination of luminal opacification and sinus wall enhancement. The cerebral venous sinuses are low-pressure endothelial lined structures, circumferentially encased by dural reflections. The lack of definite wall with muscular and fibrous layers as seen in arterial structures, precludes the distinction of luminal opacification from wall enhancement of cerebral venous sinuses on contrast enhanced MRV. Understandably this results in overestimation of the size of the sinus lumen on contrast enhanced MRV due to additional measurement of dural enhancement on either side of the luminal opacification. Our results showed an overestimation of the venous sinus diameter by 2mm on the contrast enhanced MRV compared to the TOF MRV and endoluminal measurements of IVUS (p<0.001). Consequently, sizing of the venous stent based on the contrast enhanced MRV measurements may result in erroneously large diameter stent selection by approximately 2 mm (8mm vs 10mm).

The cerebral venous sinuses are non-cylindrical triangular structures with variable luminal caliber inherent to their collapsible nature. Collapsible vessels are characterized by marked changes in their cross-sectional configuration with variations in the transmural pressure.[14] The variability of the cross-sectional contour of the venous sinus with intracranial pressure changes is well reported.[15,16] Considering non-cylindrical natures and tendency to alter cross-sectional configuration with intrinsic or extrinsic pressure changes, cerebral sinus evaluation with isolated maximum diameter may be a suboptimal choice. Simultaneous evaluation with area and perimeter will provide more robust criteria by eliminating bias related to the sinus cross-sectional alteration, which is an inevitable phenomenon in patients with IIH. Our results showed consistent overestimation by contrast enhanced MRV for all three sinus measurements including maximum diameter, area and perimeter; while time-of-flight MRV was concordant with IVUS in all three measurements.

Durst et al.[17] retrospectively reviewed the venous sinuses in 355 consecutive CT angiographic studies of general population. Authors reported a mean ± SD sinus diameter of 4.91±1.2 mm for posterior third of SSS and 5.75±1.9 mm for dominant proximal transverses sinus. Increased diameter of the transverses sinus distal to the vein of Labbe was noted with a mean ± SD of 6.22±1.8 mm. The sigmoid sinuses were not evaluated. In our cohort, the average maximum diameter of the posterior third of SSS (8.8±0.8 mm) and the proximal dominant transverse sinus (9.4±1.1 mm) on contrast enhanced MRV are significantly higher than the reported maximum sinus diameters of the general population. In contradistinction to the increased diameter of the transverses sinus distal to the vein of Labbe seen in general population, all our patients had flow limiting stenosis of the dominant distal transverse sinus immediately distal or incorporating the origin of vein of Labbe. All our patients demonstrated a post stenotic dilatation of the proximal sigmoid sinus with a mean Dmax of 10.5±1.8 mm and a mid-sigmoid sinus measuring 10±1.7 mm. The variation of findings in-terms of increased average maximum sinus diameter and flow-limiting stenosis of the distal transverse sinus at vein of Labbe and post-stenotic dilatation of the proximal sigmoid sinus in our cohort reflect the pathological sinus changes in IIH patients, the knowledge of which is critical in appropriate stent size selection. This argument is further reinforced by 23% - 93% prevalence of bilateral transverse sinus stenosis in IIH patients compared to 5% of general population, a possible pathophysiology in the IIH.

Recently, Lublinsky et al.[6] developed an algorithm for vessel cross-sectional analysis of cerebral venous sinus on contrast enhanced MRV, validated on phantom models and tested on four IIH patients with a high degree of stability and <3% of manual correction. The area of dominant transverses sinus measured by this algorithm (45±20 mm^2^) is excellent concordance with our contrast enhanced MRV area of transverse sinus (45.1±11.5 mm^2^). However, this technique needs to be further evaluated in large patient population and familiarity with the algorithm. In contrary, MRV is more widely available and validated technique with no additional infrastructure or training expenses. The maximum mean diameter of the SSS (7.3±0.8 mm) dominant transverse (7.5±1.2 mm) and sigmoid sinus (8.8±1.8 mm) of our study population are similar to the maximum endoluminal width measured on the morphometric analysis of the cerebral venous sinuses (7.9±1.5 mm, 8.5±2.4 mm and 8.6±2.0mm respectively).[18] We realize this is a single center experience and recommended larger multicenter trials to support our findings.

## Conclusion

The cerebral venous sinus measurements on time-of-flight magnetic resonance venography is a reliable representation of endoluminal sinus measurement. Contrast enhanced magnetic resonance venography measurements overestimate the size of the lumen of the sinus by approximately 2mm, because it incorporates the thickness of the dural liner to the measurement. In the absence of endoluminal sinus evaluation with intravascular ultrasound, we recommend using TOF MRV for appropriate measurement and selection of venous stent diameter. We report baseline cerebral venous sinus measurements (maximum diameter, area and perimeter) in patients with idiopathic intracranial hypertension, which are different from the sinus measurements of the general population. This information can be used towards better understanding of the IIH pathophysiology, screening of the IIH patients, appropriate selection of venous sinus stent and better device innovation.

## Supporting information

S1 File SPSS analysis. (SAV)
